# Nanopore long-read RNAseq reveals transcriptional variations in citrus species

**DOI:** 10.3389/fpls.2022.1077797

**Published:** 2023-01-04

**Authors:** Xiao-Li Hu, Congjun You, Kaikai Zhu, Xiaolong Li, Jinli Gong, Haijie Ma, Xuepeng Sun

**Affiliations:** ^1^ Collaborative Innovation Center for Efficient and Green Production of Agriculture in Mountainous Areas of Zhejiang Province, College of Horticulture Science, Zhejiang A&F University, Hangzhou, Zhejiang, China; ^2^ Key Laboratory of Quality and Safety Control for Subtropical Fruit and Vegetable, Ministry of Agriculture and Rural Affairs, Zhejiang A&F University, Hangzhou, Zhejiang, China; ^3^ Co-Innovation Center for Sustainable Forestry in Southern China, Nanjing Forestry University, Nanjing, Jiangsu, China

**Keywords:** citrus, ONT RNAseq, isoform, alternative splicing, transcript

## Abstract

The number of studies on plant transcriptomes using ONT RNAseq technology is rapidly increasing in recent. It is a powerful method to decipher transcriptomic complexity, particularly alternative splicing (AS) event detection. Citrus plants are the most important widely grown fruit crops. Exploring different AS events in citrus contributes to transcriptome improvement and functional genome study. Here, we performed ONT RNAseq in 9 species (*Atalantia buxifolia*, *Citrus clementina*, *C. grandis*, *C. ichangensis*, *C. reticulata*, *C. sinensis*, *Clausena lansium*, *Fortunella hindsii*, and *Poncirus trifoliata*), accompanied with Illumina sequencing. Non-redundant full-length isoforms were identified between 41,957 and 76,974 per species. Systematic analysis including different types of isoforms, number of isoforms per gene locus, isoform distribution, ORFs and lncRNA prediction and functional annotation were performed mainly focused on novel isoforms, unraveling the capability of novel isoforms detection and characterization. For AS events prediction, A3, RI, and AF were overwhelming types across 9 species. We analyzed isoform similarity and evolutionary relationships in all species. We identified that multiple isoforms derived from orthologous single copy genes among different species were annotated as enzymes, nuclear-related proteins or receptors. Isoforms with extending sequences on 5’, 3’, or both compared with reference genome were filtered out to provide information for transcriptome improvement. Our results provide novel insight into comprehending complex transcriptomes in citrus and valuable information for further investigation on the function of genes with diverse isoforms.

## 1 Introduction

Citrus fruits have high nutritional value and appetizing flavor. They comprise one of the most important types of fruit crops, and are wildly cultivated in more than 114 countries all around the world (Talon and Gmitter, 2008). Sweet orange (*Citrus sinensis*), mandarin (*Citrus reticulata*), pummelo (*Citrus grandis*), grapefruit (*Citrus paradisi*), and lemon (*Citrus limon*) are the top 5 cultivated citrus species (Wang et al., 2018). Due to the remarkable progress in genomics of citrus has been made, genome sequencing of 130 accession of various citrus species has been released including several key species such as Clementine mandarin, sweet orange, pummelo, citron, Ichang papeda, and Atalantia (Wang et al., 2017; Wu et al., 2018). It is an invaluable reservoir for genes that can be used to understand fruit development, metabolism of sugar or acid, and stress responses as well as for fruit crop improvement.

Alternative splicing (AS) is a ubiquitous process to generate different transcripts from the same gene, which modulates the diversity of mRNA and gene expression (Laloum et al., 2018; Li et al., 2022). Four major types of AS can be classified including intron retention (IR), alternative 3’ splice site (A3), alternative 5’ splice site (A5), and exon skipping (ES) (Hu et al., 2022). In plants, AS regulates plant development and stress responses. For instance, flowering time is tightly regulated by AS, and such critical development in plants is related to the transcription of FLOWERING LOCUS C (FLC), which is modulated by different isoforms of its anti-sense lncRNA (Marquardt et al., 2014). In addition, when *Arabidopsis* seedlings were treated with abscisic acid, AS patterns were dramatically changed as manifested by the increased number of uncanonical splicing sites (Zhu et al., 2017). The advent of high-throughput sequencing of RNA (RNAseq) has allowed us to explore the complexity of transcriptomes in different tissues and under certain conditions (Dong and Chen, 2013). However, the technologies are almost based on short-reads, which cause difficulty in computation as it is often hard to distinguish short reads derived from isoforms, particularly in plant genomes for which whole genome duplication occurred frequently and further blurred read assignment (Bernard et al., 2014; Mehrotra and Goyal, 2014).

Although short-read sequencing technologies are still the dominant method in transcriptomic studies, long-read sequencing technologies are increasingly becoming the powerful standard approach for *de novo* transcriptome assembly, isoform expression quantification as well as AS detection, which has greatly improved the study of transcriptome complexities. Currently, the newly emergent sequencing methods including Pacific Bioscience (PacBio) long-read sequencing technology and Oxford Nanopore Technologies (ONT), both provide high throughput full-length transcript sequences (Rhoads and Au, 2015; Bayega et al., 2018). Nanopore sequencing occurs in a flow cell, single-stranded DNA or RNA molecules can be sequenced through the nanopore by monitoring the change in the ionic current measured by a sensor (Van Dijk et al., 2018). ONT shows several advantages over other methods, such as extremely long sequencing reads, greater flexibility in throughput and portability of sequencing instruments, etc (Deamer et al., 2016). This approach has been successfully implemented to explore the composition of plant transcriptomes in rose (Li et al., 2020), *Gnetum luofuense* (Hou et al., 2021), and oilseed rape (Li et al., 2022). Here, we have used ONT for transcriptome sequencing of 9 citrus species and their close relatives, including *Atalantia buxifolia*, *Citrus clementina*, *C. grandis*, *C. ichangensis*, *C. reticulata*, *C. sinensis*, *Clausena lansium*, *Fortunella hindsii*, and *Poncirus trifoliata*. In addition, Illumina sequencing was also conducted. This study enables us to explore the diversity and complexity of transcriptomes among different species and provides valuable resources for future research on citrus.

## 2 Materials and methods

### 2.1 Plant material and RNA extraction

Young leaves of each species were collected and immediately frozen in liquid nitrogen. RNAprep Pure Plant Kit (Tiangen, China) was used for total RNA extraction. Genomic DNA contamination was removed by DNase treatment (Rapid Out DNA Removal Kit, Thermo Scientific, Germany). Final RNA quality and integrity were assayed using the Agilent 2100 Bioanalyzer (Agilent Technologies, Santa Clara, CA, USA) following the manufacturer’s instructions.

### 2.2 Library preparation and sequencing

For ONT transcriptome sequencing, the Oxford Nanopore Technologies kit (SQK-PCS109) was used for full-length cDNA library preparation. Sequencing was performed on the PromethION 24 platform using flow cells (PAE33370). Guppy software (Oxford Nanopore) was used for base calling. The NanoFilt tool in the Nanopack package was used to filter and preserve the sequences with length > 100 bp and quality > 7, which were recognized as clean reads for subsequent analysis. For Illumina sequencing, cDNA libraries were constructed using the NEBNext Ultra RNA Library Prep Kit for Illumina (New England Biolabs, Beverly, MA, USA) following the manufacturer’s protocol. The libraries were sequenced on an Illumina NovaSeq platform with the paired-end mode.

### 2.3 Nonredundant full-length reads identification

To generate the full-length isoform, Pychopper v2 (https://github.com/nanoporetech/pychopper) was used to identify full-length reads, which were then mapped to the reference genome of each species respectively using Minimap2 v2.17 (Li, 2018). Primarily mapped reads were obtained to remove redundancy using cDNA Cupcake (https://github.com/Magdoll/cDNA_Cupcake) with default parameters (i.e., identity < 0.9 and coverage < 0.85). Additionally, we filtered out 5’ degraded reads to obtain final non-redundant reads. Non-redundant reads were annotated using Gffcompare (Pertea and Pertea, 2020) to distinguish different types of isoforms.

### 2.4 Functional annotation of novel isoforms

Open reading frames (ORFs) of isoforms were predicted using TransDecoder (Haas et al., 2013) based on nucleotide composition, ORF length, and log-likelihood score. Novel isoforms containing complete ORFs were extracted. The resulting sequences were annotated by using Mercator4 v5 (Schwacke et al., 2019). These collapsed nucleotide sequences were submitted to the online server and the resulting files were downloaded.

### 2.5 LncRNA and AS prediction

Four different software were used to predict lncRNA, including Coding Potential Calculator (CPC2) (Kang et al., 2017), Coding–Non-Coding Index (CNCI) (Sun et al., 2013), PLEK (Li et al., 2014), and Pfam (Mistry et al., 2021) with the default setting. Finally, isoforms identified by all four tools and larger than 200 bp without coding potential were selected as candidate lncRNAs. Venn diagrams were drawn by R. Suppa software (Trincado et al., 2018) and utilized to define AS events with default parameters.

### 2.6 Quantification of gene expression

For Illumina sequencing, STAR 2.7.10 (https://github.com/alexdobin/STAR) and featureCounts (Liao et al., 2014) were used for reads alignments and gene/transcript counting, respectively. Gene expression levels were estimated by transcripts per million (TPM). For ONT RNAseq, the number of transcripts was figured using Salmon (Patro et al., 2017), and gene expression levels were calculated by TPM. The correlation between Illumina and ONT RNAseq was calculated using Pearson correlation coefficient.

### 2.7 Determination of common isoforms

To determine the common overlap of isoforms among 9 species, we ran blastn v2.13.0 (Camacho et al., 2009) on our own server with E-value < 1E-10. Blast databases were constructed using non-redundant reads of *C. sinensis* and isoforms of additional eight species were blasted against the database. The best hit for database entry of each species was kept to analyze the common overlap of isoforms. Results were visualized by R.

### 2.8 Identification of orthologous genes

The primary protein sequences of 9 species were used as input to construct ortholog groups and species tree using Orthofinder (Emms and Kelly, 2019). Gene synteny analysis and image of orthologous genes among 9 species were generated using the jcvi program (https://github.com/tanghaibao/jcvi) with the default parameters.

## 3 Results

### 3.1 Overview of ONT transcriptome sequencing

We selected 9 species including *A. buxifolia*, *C. clementina*, *C. grandis*, *C. ichangensis*, *C. reticulata*, *C. sinensis*, *C. lansium*, *F. hindsii*, and *P. trifoliata* for this study. Leaves for each species were harvested to prepare RNA libraries for ONT transcriptome sequencing. In total, the size of clean reads obtained for different species ranged from 5.59 to 6.26 gigabases (GB) (**Supplementary Table 1**). Clean reads were processed with Pychopper to identify full-length reads, resulting in between 5,030,899 and 8,319,455 full-length reads per species (**Supplementary Table 2**). We used cDNA cupcake to collapse redundant isoforms with degraded 5’ based on the genome sequence of each species. The number of isoforms was remarkably reduced, yielding 41,957 to 76,974 non-redundant isoforms in different species (**Supplementary Table 1**).

### 3.2 Isoform characterization

**Figure 1:**
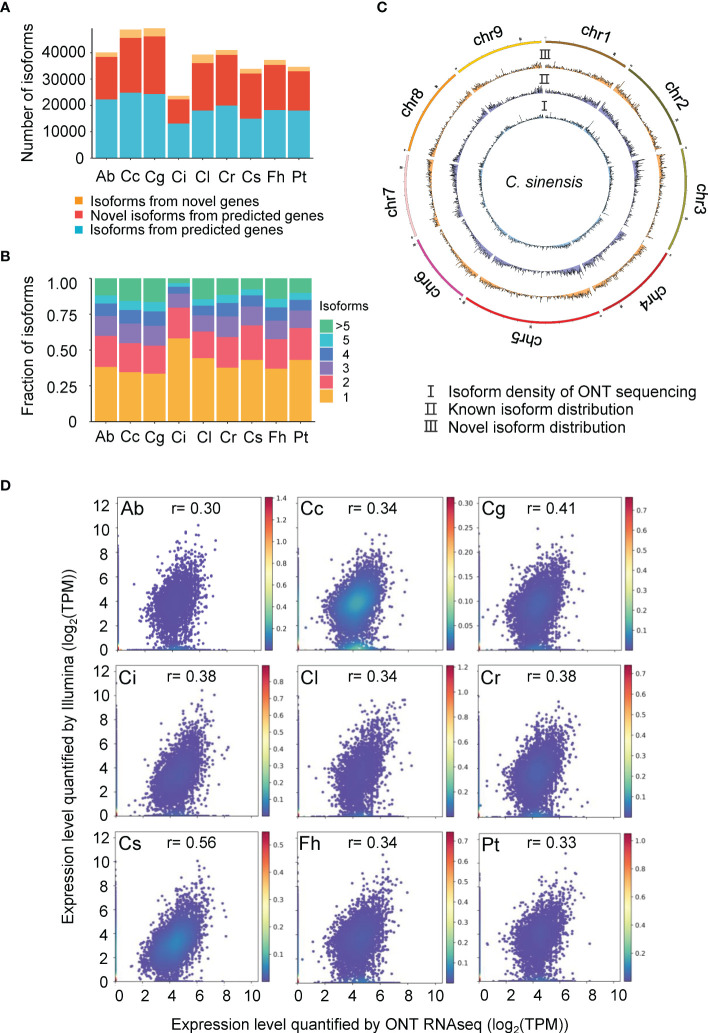
Characterization of different isoforms identified in 9 species. Ab, *Atalantia buxifolia*; Cc, *Citrus clementina*; Cg, *Citrus grandis*; Ci, *Citrus ichangensis*; Cl, *Clausena lansium*; Cr, *Citrus reticulata*; Cs, *Citrus sinensis*; Fh, *Fortunella hindsii*; Pt, *Poncirus trifoliata*. **(A)** Number of three types of isoforms (isoforms from predicted genes, novel isoforms from predicted genes, and isoforms from novel genes) identified across 9 species. **(B)** Fraction of isoforms per gene across 9 species. **(C)** Chromosomal distribution of isoforms generated by ONT RNAseq, using **(C)** sinensis as representative species. I: Isoform density of ONT sequencing; II: Known isoform distribution; III: Novel transcript distribution. **(D)** Gene expression correlations between Illumina and ONT RNAseq. Scatter plot shows gene expression for each gene determined by Illumina and ONT RNAseq for 9 species. Gene expression were given as Transcripts Per Million (TPM). Person r were calculated for each species.

ONT generates full-length reads without assembly which allows us to explore the complexity of transcriptome more accurately than does short-read sequencing. Non-redundant isoforms of the 9 species were compared with their reference annotation respectively using Gffcompare. This tool is able to report multiple types of isoforms including known isoforms and novel isoforms from predicted genes and isoforms from novel genes, and the accuracy of splicing junction sites based on ONT sequencing showed high consistency with that predicted from Illumina sequencing. Around 30% of isoforms were reported as novel in all species (**Figure 1A**), which affirms that ONT RNAseq is a powerful technology to detect novel isoforms in transcriptomes. It is worth noting that isoforms characterized from novel genes account for a small proportion of transcriptomes (**Figure 1A**), however, the rate varies among species and negatively correlates with the completeness of the genome assembly.

We also determined the number of isoforms per gene, the results showed that single isoform per gene was prevalent among the transcriptome of 9 different species, which represents 34-58% of their transcriptomes (**Figure 1B**). In addition, the isoform density obtained from ONT RNAseq was shown (**Figure 1C** and **Supplementary Figure 1**). Since some species were without chromosome-level genome assembly, the top 50 largest fragments were used for plotting.

### 3.3 Quantification of gene expression

To quantify gene expression, we compared RNAseq data of 9 species generated by ONT and Illumina. Standard gene quantification tools do not apply for full-length reads, we chose salmon to estimate the gene expression using reads generated through ONT RNAseq by TPM. For Illumina sequencing, TPM values of each gene were obtained as well. When comparing ONT RNAseq and Illumina gene expression quantification, the highest correlation value was observed in *C. sinensis* (**Figure 1D**).

### 3.4 Prediction and functional annotation of ORF and lncRNA

**Figure 2:**
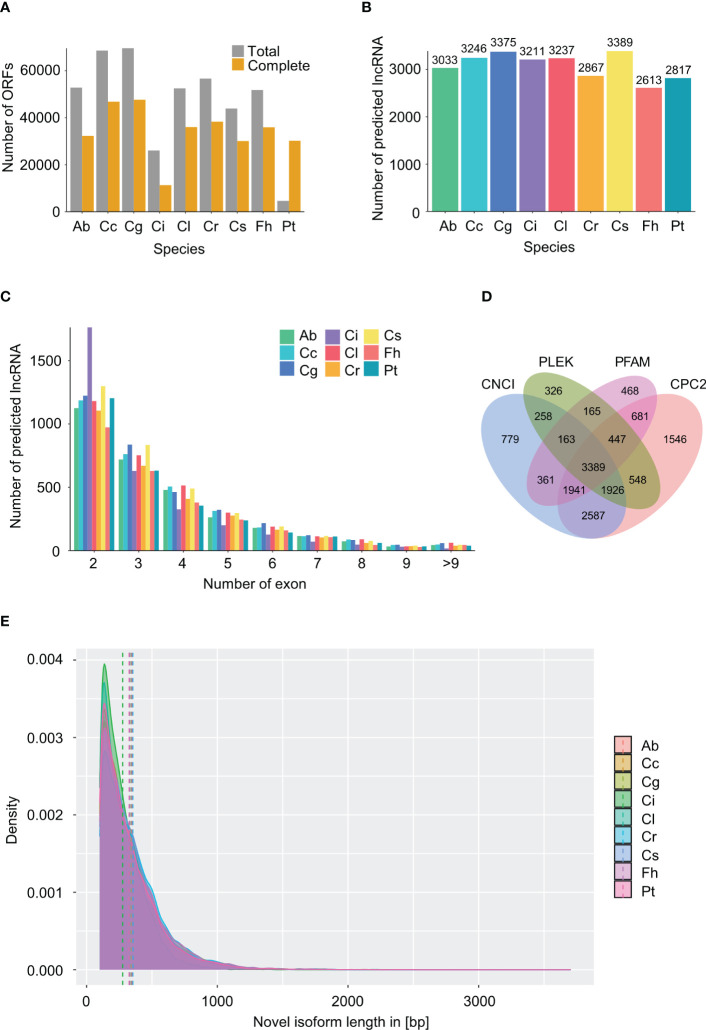
Information on identified ORFs and lncRNA. *Atalantia buxifolia*; Cc, *Citrus clementina*; Cg, *Citrus grandis*; Ci, *Citrus ichangensis*; Cl, *Clausena lansium*; Cr, *Citrus reticulata*; Cs, *Citrus sinensis*; Fh, *Fortunella hindsii*; Pt, *Poncirus trifoliata*. **(A)** Number of total and complete ORFs within novel isoforms in 9 species predicted using TransDecoder. **(B)** Number of lncRNA predicted by all four tools (CNCI, CPC2, PFAM and PLEK) in 9 species. **(C)** Number of exons within each predicted lncRNA in 9 species. Single exon lncRNAs were excluded. **(D)** Venn diagram of identified lncRNAs by using four tools (CNCI, CPC2, PFAM and PLEK) in Citrus sinensis. **(E)** Length distribution of novel isoforms with complete ORFs for 9 species. Dashed lines show the mean length of isoforms for each species.

To further understand the biological roles of novel isoforms, we firstly created subsets of novel isoforms (including novel isoforms from predicted genes and isoforms from novel genes) for each species. Open reading frames (ORFs) were predicted using TransDecoder, and 26,058 to 69,640 ORFs were identified, of which 11,300 to 47,648 complete ORFs were predicted in 9 species (**Figure 2A**). Length distribution of novel isoforms containing complete ORFs was determined, showing that most isoforms were less than 1,000 bp (**Figure 2E**). The largest mean length of novel isoforms encoding complete ORFs was 353 bp in *C. reticulata*, followed by 347 bp in *F. hindsii*, which were far shorter than those of non-redundant reads. Furthermore, we screened novel isoforms for putative long non-coding RNAs (lncRNAs) using CPC2, CNCI, PLEK, and PFAM databases, a total of 2,613 to 3,389 lncRNAs were predicted across 9 species (**Figure 2B**). Furthermore, the number of exons within lncRNAs was counted. LncRNA with 2 exons were overwhelming in all species (**Figure 2C**). The results of lncRNA prediction by individual method were shown in **Figure 2D** and **Supplementary Figure 2**.

Functional annotation of novel isoforms was performed using Mercator4. Mercator4 is a user-friendly, plant-specific biological function annotation tool. The number of annotated sequences in each “bin” for 9 species were collected and shown in **Supplementary Figure 3**. The relative distribution is similar among the 9 species. Major bins were described as enzymes, biosynthesis and modification of RNA and protein, and solute transport.

### 3.5 Evaluation of isoforms among species

**Figure 3:**
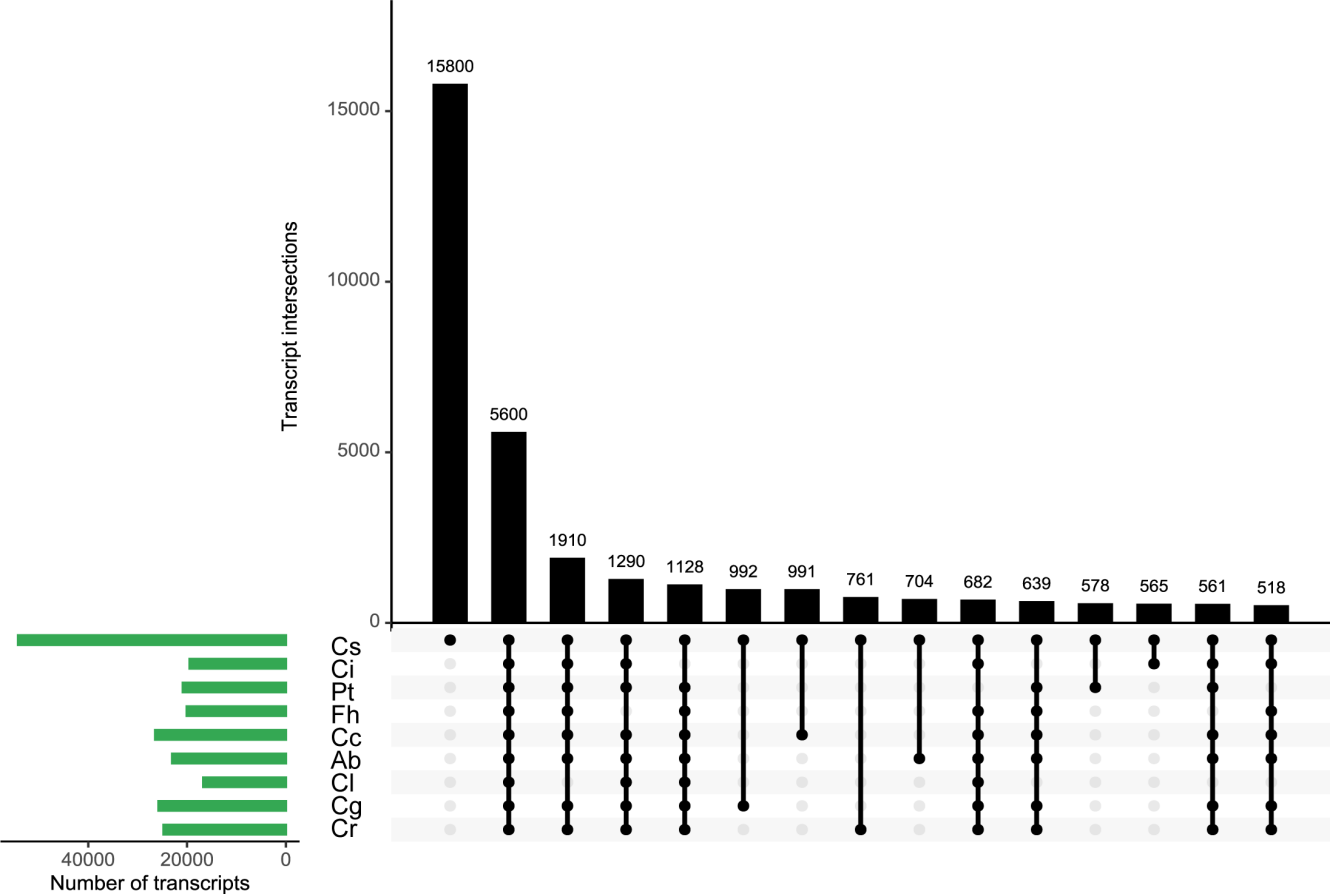
Identification of common and specific transcripts across 9 species. *Atalantia buxifolia*; Cc, *Citrus clementina*; Cg, *Citrus grandis*; Ci, *Citrus ichangensis*; Cl, *Clausena lansium*; Cr, *Citrus reticulata*; Cs, *Citrus sinensis*; Fh, *Fortunella hindsii*; Pt, *Poncirus trifoliata*. Database was built using non-redundant reads of Citrus sinensis, sequence similarities were determined using blastn search. The best hit for entry of each species was collected. The 15 largest categories were visualized in an UpSet plot. The number of transcripts in each category was presented in the top barplot. Entry size of different species was shown in the left barplot. Black dots and vertical lines in the lower panel indicate the species to which transcripts belong.

To identify cultivar-specific transcripts, the non-redundant isoforms of *C. sinensis* were used as a blast database and sequences of the remaining eight species were searched against it. The best hit for database entry of each cultivar was selected and the common overlap with all other cultivars was determined. In total, 54,222 transcripts of *C. sinensis* were used to build the database, of which about 5,600 were highly similar to isoforms from the other eight cultivars (**Figure 3**). However, 15,800 transcripts were unique to *C. sinensis* (**Figure 3**), suggesting the potential genetic variations existing between *C. sinensis* and other species.

### 3.6 Analysis of alternative splicing events

**Figure 4:**
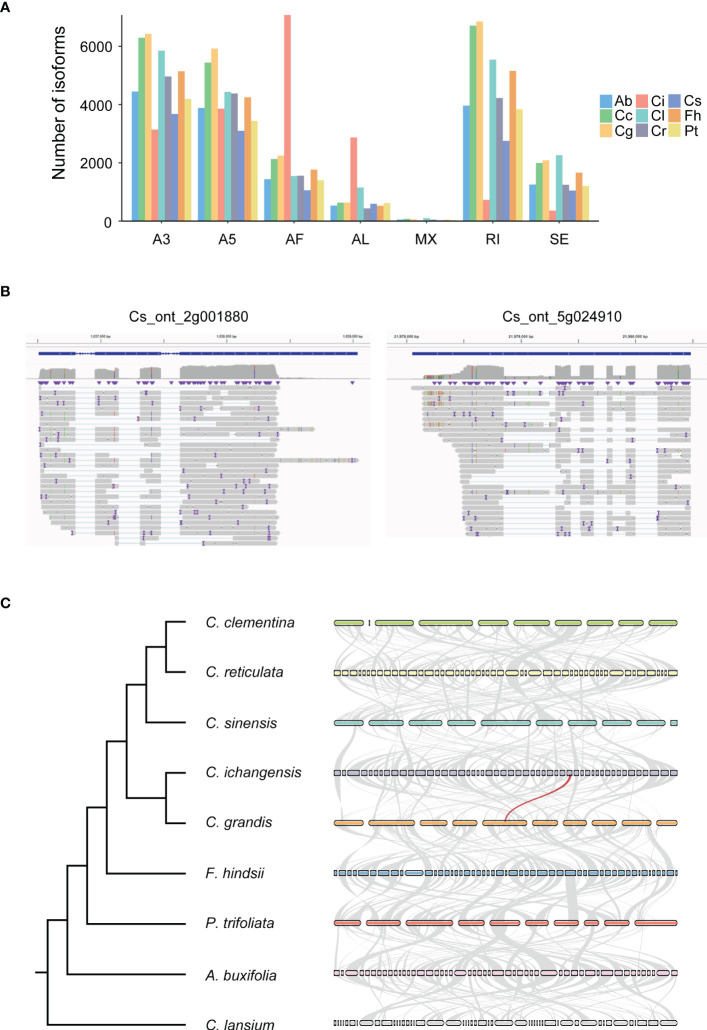
Characterization of different types of alternative splicing (AS) events and evolution of orthologous genes in 9 species. Ab, *Atalantia buxifolia*; Cc, *Citrus clementina*; Cg, *Citrus grandis*; Ci, *Citrus ichangensis*; Cl, *Clausena lansium*; Cr, *Citrus reticulata*; Cs, *Citrus sinensis*; Fh, *Fortunella hindsii*; Pt, *Poncirus trifoliata*. **(A)** AS events were predicted using SUPPA. A3, Alternative 3’ splice sites; A5, Alternative 5’ splice sites; AF, Alternative first exons; AL, Alternative last exons; MX, Mutually exclusive exons; RI, Retained intron; SE, Skipping exon. **(B)** The full-length reads representing the complex isoforms were mapped to *Cs_ont_2g001880* and *Cs_ont_5g024910*, which illustrates diverse isoforms can be found by ONT RNAseq. The result was visualized by IGV. **(C)** A species tree generated by Orthofinder is shown on the left. Synteny analysis of ortholog genes among all species is shown on the right. The top 50 largest genomic fragments were used to construct the synteny network. The light grey lines indicate collinear blocks, the red line indicates ortholog single copy gene has highly abundant AS variation.

We identified seven types of AS events including A3, A5, AF (Alternative first exon), AL (Alternative last exon), MX (Mutually exclusive exons), RI, and SE in 9 species using SUPPA software. The most abundant events were A3 (28.6%, 28.0%, 29.5%, 30.0% and 28.5% in *A. buxifolia*, *C. lansium*, *C. reticulata*, *C. sinensis* and *P. trifoliata*), RI (28.8%, 28.3%, and 27.8% in *C. clementina*, *C. grandis*, and *F. hindsii*) and AF (39.2% in *C. ichangensis*) (**Figure 4A**). In contrast, the least abundant event was MX in all species (0.1% to 0.5%).

To explore the variation on the frequency of AS events in orthologous genes across 9 species, ortholog groups were constructed using Orthofinder, and we focused on 4,827 ortholog groups containing single copy genes originating from all species. Different transcripts of these single copy genes were identified according to ONT RNAseq data. Based on transcript numbers, we screened out the largest ten ortholog groups comprising between 128 to 248 transcripts, in which protein sequences of *C. sinensis* were used as representative for functional annotation (**Supplementary Table 3**). Unsurprisingly, these proteins are enzymes, nuclear-related proteins and receptor. Their multiple complex isoforms determine the varying functions. Representative gene locus with high isoform diversification in *C. sinensis* was illustrated in **Figure 4B** and syntenic network for single copy genes of the 9 species were shown in **Figure 4C** and **Supplementary Table 4**.

### 3.7 Reference genome improvement

Since ONT RNAseq enables to identify features like TSS (Transcription Start Site) and TES (Transcription End Site), we screened the representative isoforms with the longest ORFs identified by Gffcompare and discovered 1,034 to 4,467 isoforms with complete TSS and TES in all species. This result improved the accuracy of genome annotation and may facilitate future study in respective species (**Supplementary Table 5**).

## 4 Discussion

As plant research has entered the genomic era, numbers of genome sequenced plant species have grown up with an exponential rate over the past two decades with no slowdown in sight. Citrus is one of the most widely grown fruit crops (Sun et al., 2022). Several representative citrus genomes have been released, which accelerate genetic studies and gene functional exploration. In addition, decreasing the cost of high-throughput sequencing technology has extended our understanding of the transcriptome landscape in citrus. For instance, high-spatiotemporal-resolution transcriptomes were used to profile *Citrus sinensis* fruit development and ripening based on four fruit tissue types and six fruit development stages, which provides insights into the molecular networks from young fruits to ripe fruits (Feng et al., 2021). In another study, RNAseq analysis was performed to identify 311 differentially expressed genes (DEGs) in the bagged pomelo (*Citrus grandis*) and controls during five fruit developmental stages. Most DEGs were involved in the carotenoid pathway and lycopene accumulation. The results facilitated the improvement of fruit nutritional quality (Jiang et al., 2022). However, these studies used short-read sequencing technology, which required transcriptome assembly and failed to obtain full-length transcripts and AS events. The emergent long-read sequencing technology provides a significant chance to carry out studies on full-length transcriptome and complex AS events. As a matter of fact, comparative transcriptome analysis is a powerful approach to gain insights into gene function and evolution (Xu et al., 2022). In this study, we selected 9 species with reference genomes respectively. ONT RNAseq was applied to generate long-length reads, besides systematic analysis of genetic changes in transcriptome, we also performed comparative transcriptome analysis and inferred divergence and evolution of transcripts.

ONT RNAseq empowered the discovery of novel AS, as generating a huge number of long reads without transcriptomic assembly. Between 5.59 and 6.26 GB of cDNA were sequenced for each species resulting in from 5,181,086 to 8,319,455 full-length reads (**Supplementary Table 2**). Additionally, the published citrus genomes were assembled by using short-length sequencing technology, their annotations are not quite thorough. Even for the well-annotated rice transcriptome, about 17% of isoforms could not find the functional description and were considered as novel isoforms when long-read sequencing was fulfilled (Schaarschmidt et al., 2020). Indeed, a large fraction of novel isoforms were identified by using Gffcompare software, the ratio is between 25.0% and 34.8% among 9 species.

For AS events detection, A3 events showed the largest proportion in the current study, followed by RI and AF. The three types of AS events are overwhelming in all kinds of AS events. ONT RNAseq has been successfully applied to other species to detect AS events. In resynthesized and natural *B. napus*, a total of 9,296 and 10,820 AS events were identified (Li et al., 2022), suggesting that long-read sequencing technology can serve as an efficient method for transcriptome study and functional genomics in different plant species.

Long-read sequencing also showed advantages in identifying lncRNAs. Complete ORFs within novel isoforms were predicted in 9 species. Four tools were used for the identification of lncRNAs. A total of 2,613 to 3,389 lncRNAs were predicted by all four approaches, which suggested the robustness of lncRNA prediction using ONT RNAseq. Functional annotation of novel isoforms revealed that sequences classified into groups related to enzyme classification, biosynthesis, modification of RNA and protein, and solute transport accounted for an overwhelming proportion. The results indicated that copious transcripts involve in these pathways remaining to be studied.

To explore common isoforms among 9 species, we used non-redundant isoforms of *C. sinensis* to build a database for blast searches of the rest species. By this method, we identified common and species-specific isoforms in our datasets. A total of 15,800 *C. sinensis* specific isoforms were yielded, followed by 5,600 common isoforms in all species. The analysis illustrated that certain transcriptomic variation and consistency exist among 9 species. To deepen the understanding of evolutionary relationship of AS, we performed phylogenomic analysis and filtered out single copy ortholog groups. Numbers of AS events derived from such groups were counted, top ten were selected for functional annotation which represented highly diversity of AS events. These genes expressing complex isoforms were characterized as enzymes, nuclear-related proteins and receptors. Our results recapitulated the finding in human B1a cells sequenced using Nanopore long-read RNAseq, in which several B cell-specific surface receptors expressed multiple complex isoforms were identified to confirm the exceptional transcriptional diversity (Byrne et al., 2017).

We assessed the correlation of gene expression quantification between ONT RNAseq and Illumina in 9 species. The highest correlation value is 0.58 found in *C. sinensis*. While for *Arabidopsis* (Cui et al., 2020) and human (Byrne et al., 2017) whose genome annotations are much better, the correlation values were greater than 0.8 in both cases. Therefore, we propose that additional efforts should be made to improve citrus genome annotation in the future. Another evidence occurred when we visualized multiple isoforms generated from gene locus *Cs_ont_5g024910*, gene structure variations were detected when comparing reference genome annotation to full-length read sequenced using ONT RNAseq (**Figure 4B**). For this reason, we tried to improve genome annotation by discovering transcripts with extending the length of 5’ or 3’ or both compared with the reference genome. As a result, we find from 1,034 to 4,467 optimized transcripts, indicating the capability of genome improvement by long-read sequencing.

## Conclusion

ONT RNAseq allows us to profile complex transcriptomes in citrus species. We discovered novel transcripts, analyzed abundance AS events and attempted to improve the reference transcriptome. To understand evolutionary variations on isoform numbers of ortholog genes across different citrus species, some enzymes and receptors were identified to show high varied number of isoforms in different species. This result can be used as a resource to explore environmental adaptation across citrus related linages.

## Data availability statement

The original contributions presented in the study are publicly available. This data can be found here: NCBI, PRJNA894942 and FigShare, 10.6084/m9.figshare.21384195.

## Author contributions

XS and X-LH conceived the project. X-LH wrote the paper. All authors contributed to the article and approved the submitted version.
