# Supplementary material for: Nanopore long-read RNAseq reveals transcriptional variations in citrus species
Source: Front Plant Sci. 2023 Jan 4;13:1077797. doi: 10.3389/fpls.2022.1077797 (PMC9845879; doi:10.3389/fpls.2022.1077797)
Supplement: Supplementary file 6 [file DataSheet_1.pdf]

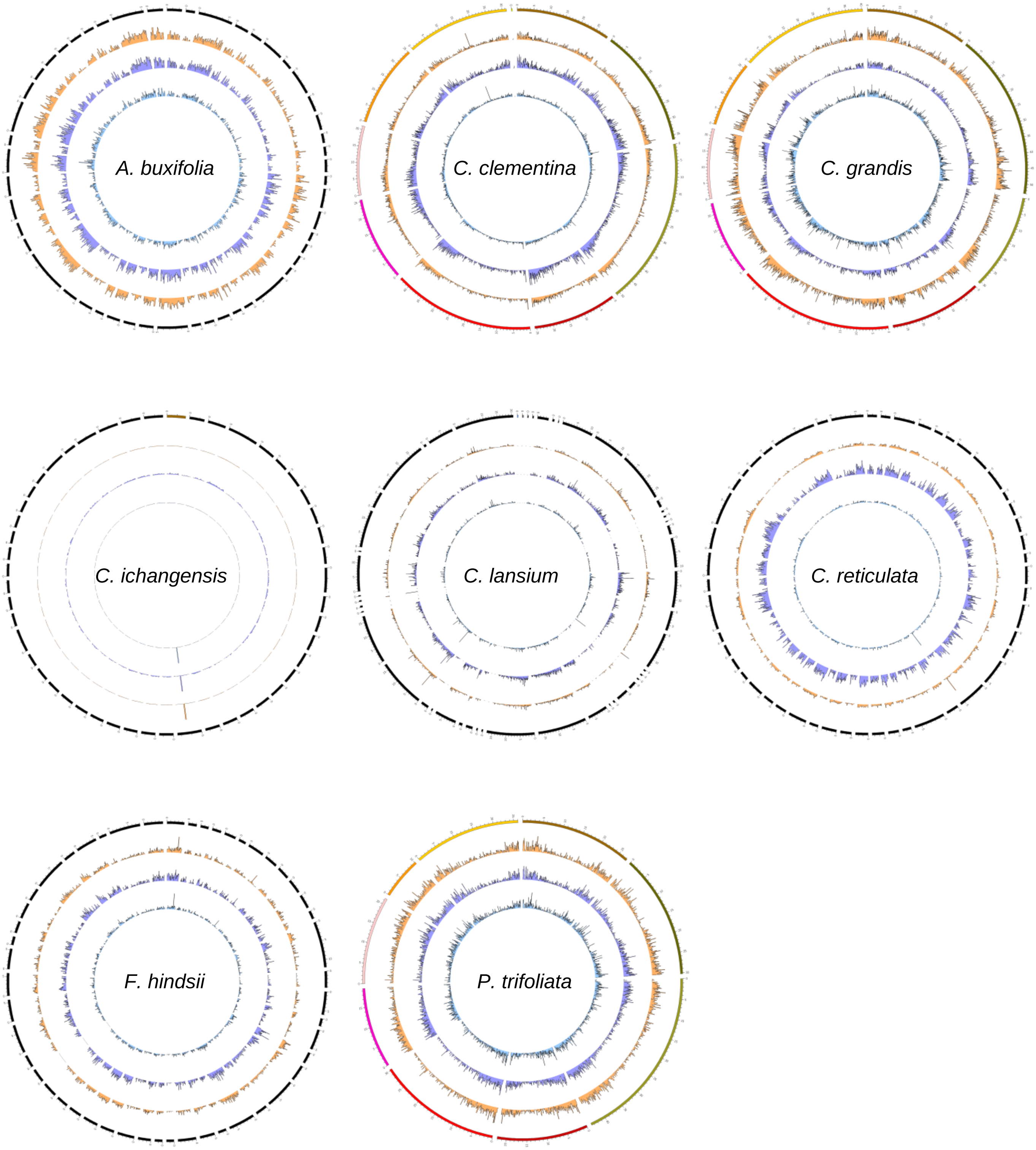


**Supplementary Figure 1. Distribution of isoforms generated by ONT RNAseq in different species**. Top 50 largest genomic fragments were selected if species without chromosomal genome assembly to draw the plots. From outside to inside, the circles present genomic fragment/chromosome, isoform density of ONT sequencing, known isoform distribution, novel transcript distribution in order. Species name shown in the center of each plot.

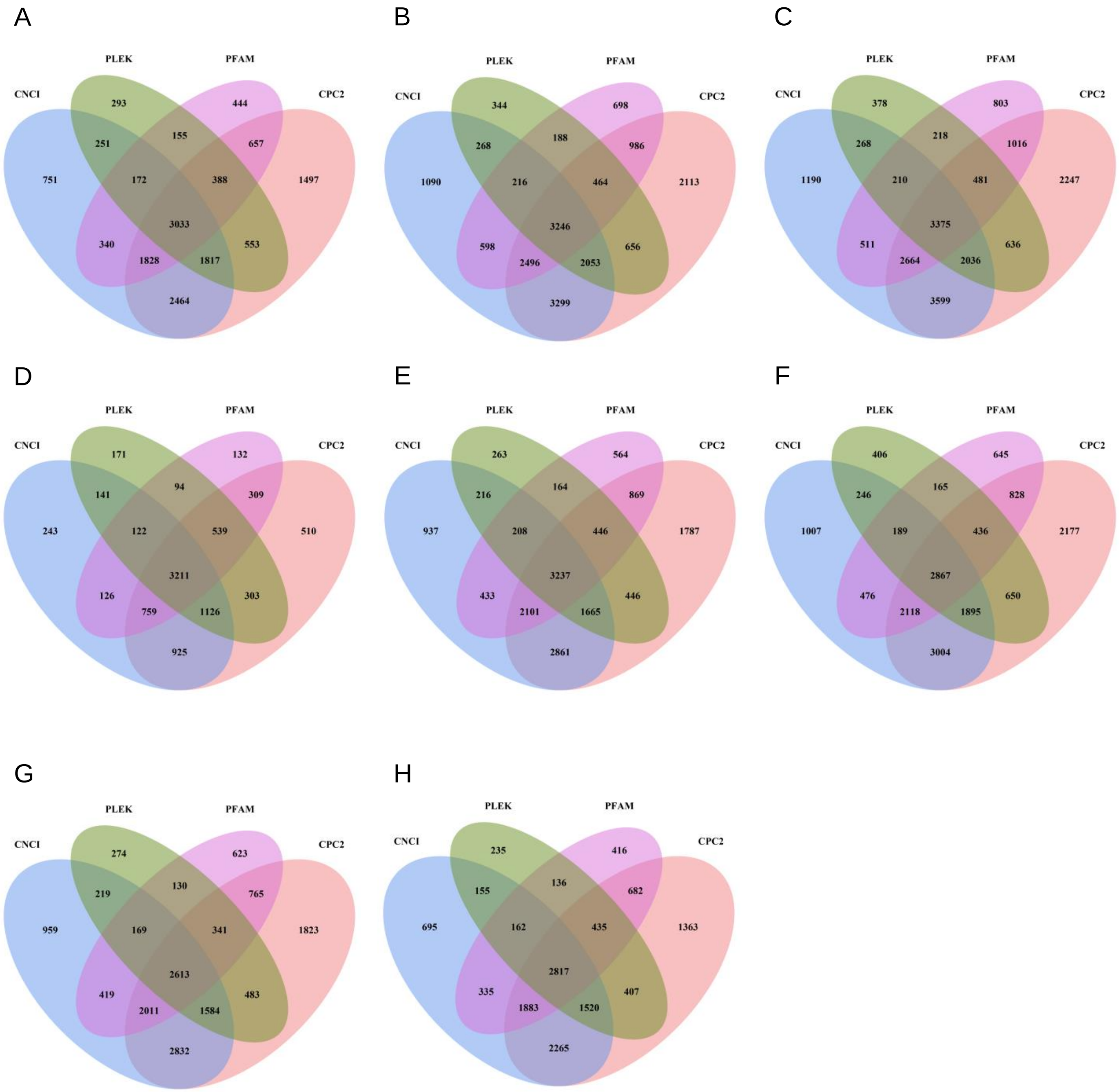


**Supplementary Figure 2. Number of predicted lncRNAs in different species.** Venn diagram of identified lncRNAs by using four tools (CNCI, CPC2, PFAM and PLEK) in 8 species. A: *Atalantia buxifolia*; B: *Citrus clementina*; C: *Citrus grandis*; D: *Citrus ichangensis*; E: *Clausena lansium*; F: *Citrus reticulata*; G: *Fortunella hindsii*; H: *Poncirus trifoliata*.

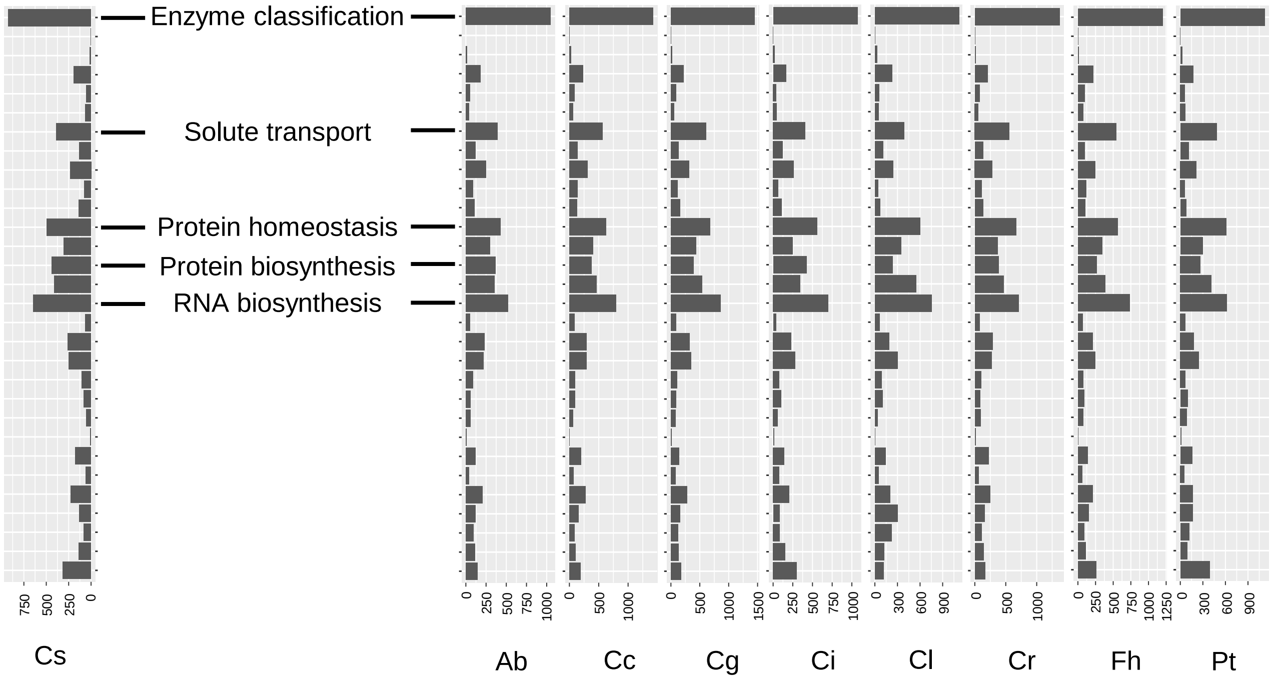


**Supplementary Figure 3. Functional annotation of novel isoforms by different bins.** Ab: *Atalantia buxifolia*; Cc: *Citrus clementina*; Cg: *Citrus grandis*; Ci: *Citrus ichangensis*; Cl: *Clausena lansium*; Cr: *Citrus reticulata*; Cs: *Citrus sinensis*; Fh: *Fortunella hindsii*; Pt: *Poncirus trifoliata*. Novel isoforms of 9 species were classified into different bins using Mercator. The names of several large bins were shown.
